# Proficiency testing for HIV, tuberculosis and malaria diagnosis in clinical laboratories in Nigeria

**DOI:** 10.4102/ajlm.v3i1.102

**Published:** 2014-10-24

**Authors:** Rosemary A. Audu, Catherine C. Onubogu, Rosemary N. Okoye, Nkiru N. Nwokoye, Chika K. Onwuamah, Adesola Z. Musa, Toyosi Y. Raheem, Maureen N. Aniedobe, Samuel J. Nduaga, Ini-Obong Essien, Emmanuel O. Idigbe

**Affiliations:** 1 Human Virology Laboratory, Nigerian Institute of Medical Research, Nigeria; 2 National Tuberculosis Reference Laboratory, Nigerian Institute of Medical Research, Nigeria; 3 Clinical Diagnostic Laboratory, Nigerian Institute of Medical Research, Nigeria; 4 Monitoring and Evaluation Unit, Nigerian Institute of Medical Research, Nigeria

## Abstract

**Background:**

Proficiency testing (PT) is a means of verifying the reliability of laboratory results, but such programmes are not readily available to laboratories in developing countries. This project provided PT to laboratories in Nigeria.

**Objectives:**

To assess the proficiency of laboratories in the diagnosis of HIV, tuberculosis and malaria.

**Methods:**

This was a prospective study carried out between 2009 and 2011. A structured questionnaire was administered to 106 randomly-selected laboratories. Forty-four indicated their interest in participation and were enrolled. Four rounds of pre-characterised plasma panels for HIV, sputum films for tuberculosis and blood films for malaria were distributed quarterly by courier over the course of one year. The results were returned within two weeks and scores of ≥ 80% were reported as satisfactory. Mentoring was offered after the first and second PT rounds.

**Results:**

Average HIV PT scores increased from 74% to 95% from the first round to the third round, but decreased in the fourth round. For diagnosis of tuberculosis, average scores increased from 42% in the first round to 78% in the second round; but a decrease to 34% was observed in the fourth round. Malaria PT performance was 2% at first, but average scores increased between the second and fourth rounds, culminating in a fourth-round score of 39%. Many participants requested training and mentoring.

**Conclusions:**

There were gross deficiencies in the quality of laboratory services rendered across Nigeria. In-country PT programmes, implemented in conjunction with mentoring, will improve coverage and diagnosis of HIV, tuberculosis and malaria.

## Introduction

The importance of quality services in healthcare laboratories in developing countries has been recognised universally.^1,2,3^ At present, the laboratory infrastructure and test quality for all types of clinical laboratories remains weak in most countries in Africa.^4,5^ Laboratories applying the principles of a quality management system (QMS) generate reliable and cost-effective results; moreover, quality management is one of the major building blocks of the accreditation process in the African region.^6,7^ It has become necessary to strengthen the capacity of clinical laboratories in order to ensure the generation of quality results that are suitable for use by clinicians and which benefit patients.

Proficiency testing (PT) is an external quality assessment (EQA) programme involving sending a panel of samples to a group of participating laboratories.^8^ Although the organisers issuing the panels know the result, the participating laboratory personnel do not. PT verifies that laboratories are proficient in their testing process and can obtain accurate and reliable results.^6^ Comparison of results between groups of laboratories may also be used to validate a particular measurement process. As beneficial as the PT programmes may be, they are not readily available to many laboratories in developing countries. Some of the limitations to local laboratories' enrolment in foreign programmes are the high cost, challenges of transportation with respect to country regulations, suitable means of transport of specimens to sites, difficulty in interpretation of PT results and the absence of technical support with regard to identifying and correcting causes of poor performance.^6^

Since laboratory-confirmed diagnosis of HIV, tuberculosis (TB) and malaria is essential to public health prevention and support services, accurate and reliable laboratory results are critical.^9^ In addition, the World Health Organization's Regional Office for Africa (WHO AFRO) has recommended that National Public Health Reference Laboratories develop and implement a QMS,^2^ including participation in an EQA scheme.^7^ These reference laboratories, according to WHO AFRO, should, in turn, provide national EQA programmes to other laboratories within the country.^7^ As a result of the challenges encountered by laboratories, a national PT feasibility study for HIV, TB and malaria was undertaken at both public and private health laboratories in Nigeria. This study was conducted to evaluate the level of implementation of QMS in Nigeria and to assess the proficiency of laboratories in the diagnosis of HIV, TB and malaria.

## Research method and design

This was a prospective study carried out in two phases between 2009 and 2011. The first phase was a questionnaire survey to provide baseline information on quality practices in laboratories, whilst the second phase was the provision of PT services.

### Phase 1: Questionnaire survey

In the first phase, six states were selected at random from each of the six geopolitical zones of Nigeria. Six focal persons who were laboratory state coordinators were then identified and recruited to serve as zonal coordinators. Zonal coordinators identified 20 laboratories, each from different local government areas within the six states. Structured questionnaires, adapted from the WHO template,^10^ were developed and field tested prior to distribution of the survey (Box 1). The questionnaire had five sections, including general questions about the laboratory, specific questions about HIV, TB and malaria units and questions on general quality issues. The laboratory head and respective heads of each unit completed the questionnaires. The questionnaires were sent to the zonal coordinators to administer to the 120 laboratories, 106 of which consented to participate and returned completed questionnaires. Data entry and analysis were performed using the FileMaker Pro v10 (2009) database and Microsoft® Excel, respectively.

BOX 1 Needs assessment questionnaire for the national external quality assessment programme for HIV, tuberculosis and malaria.

**Section A – To be completed by head of the laboratory General (Tick all that apply)**

1. What are your contact details?

**Table:** 

Laboratory	Contact Person
Name of Lab ………………………	Name ………………………………….
Address ………………………………	Address ……………………………….
Telephone ……………………………	Telephone …………………………….
E-mail …………………………………	E-mail ………………………………….
L.G.A …………………………………	
State ………………………………….
2. Type of laboratory (tick all that applies)?
  (a) Government
  (b) Private
  (c) University
  (d) NGO
  (e) Standalone
  (f) Hospital based
  (g) Mobile
  (h) Others (specify) ……………………………………………………………….
3. Number of laboratory staff?
  PhD …………
  MBBS …………
  MSc …………
  Med Lab Scientist …………
  Lab technician …………
  Support staff …………
4. Does your laboratory have:
  (4.1) Standard Operating Procedures for each assay?
  (a) Yes
  (b) No
  (4.2) Infectious waste disposal guidelines?
  (a) Yes
  (b) No
5. Does your lab have reliable internet services?
  (a) Yes
  (b) No
6. Does your lab have back up power source (e.g. generator)?
  (a) Yes
  (b) No
7. (7.1) How are laboratory results stored (e.g. Register, file, electronic database etc)? ……………………………………………………….
  (7.2) For how long are these results stored? ………. (months/years). Tick the correct unit.
8. Do you have a system for validating results before being released?
  (a) Yes
  (b) No
9. Do you have a system and criteria for the evaluation and selection of suppliers of kits, reagents and materials?
  (a) Yes
  (b) No
10. (10.1) Do you transport samples to other labs
  (a) Yes
  (b) No
  (10.2) For what assays (specify)? …………………………………………….
  (10.3) How far is the receiving lab from your facility by road (e.g. 10 min walk, 2 hr drive)? …………………………………………………………….
  (10.4) What are the conditions for transporting samples
  (a) Fresh
  (b) Frozen and on ice
  (c) Dry ice
  (d) Liquid nitrogen
  (10.5) What are the methods for shipping samples to the receiving lab?
  (a) By road
  (b) By air
  (c) By courier
  (d) Others (specify ) ………………………………………………………….
  Do you have supplies forecast and inventory management system in your lab?
  (a) Yes
  (b) No
11. What are the waste disposal methods you use? (tick all that apply)
  (a) Biohazard bags
  (b) Autoclaves
  (c) Incinerator/Burning
  (d) Separate liquid waste treatment
12. What is the safety equipment you use (tick all that apply)
  (a) Gloves
  (b) Lab coats
  (c) Safety cabinets
  (d) Goggles
  (e) Face masks
  (f) Respirators (N95)
13. Do you have any preventive maintenance agreement for your equipment?
  (a) Not applicable
  (b) Daily
  (c) Weekly
  (d) Monthly
  (e) Quarterly
  (f) Don’t have
  (g) Others (specify) ………………………………………………………….

**Section B – to be completed by head of the HIV unit HIV diagnosis (Tick all that apply)**

1. Which of these tests do you perform?
  (a) Rapid EIA
  (b) ELISA
  (c) Western Blot
  (d) Nucleic-acid testing
2. What algorithm do you use for your diagnosis?
  (a) Serial
  (b) Parallel
  (c) Not applicable
  (d) Others (specify) ………………………………………………………….
3. What is your test turn-around time for HIV diagnosis?
  ………….Days/hrs (please circle the appropriate unit)
4. What is the average number of tests you perform monthly?
  (a) Less than 50
  (b) 55–99
  (c) 100–199
  (d) 200–499
  (e) 500 and more
5. How many non-laboratory staff has been trained in HIV diagnosis between January – December 2008? ……………………………………….
6. (6.1) How many laboratory personnel have been retrained in HIV diagnosis between January – December 2008? …………………………
  (6.2) What kind of training was done?
  (a) In-house
  (b) Local
  (c) International
7. How are samples stored?
  (a) Room temp
  (b) 2–8 °C
  (c) -20 °C
  (d) -70 °C
8. For how long are they stored before analysis? …………………. days.
9. (9.1) Have you registered for any external quality assurance programme for HIV diagnosis?
  (a) Yes
  (b) No
  (9.2) If yes, which is it …………………………………………………………. ?
  And what assay …………………………………………………………………… ?
  (9.3) If no, would you want to register for a national external quality assurance programme?
  (a) Yes
  (b) No
10. (10.1) Do you have any in-house quality control and assurance measures?
  (a) Yes
  (b) No
  (10.2) If yes, list all?
  (a) …………………………………………………………………………………
  (b) …………………………………………………………………………………
  (c) …………………………………………………………………………………. ………………………………………………………………………………………

**Section C – to be completed by head of the TB unit TB diagnosis (Tick all that applies)**

1. Which of these tests do you perform?
  (a) AFB Direct Smear Microscopy
  (b) NaOH/NALC concentrated method
  (c) Fluorescent microscopy
  (d) Bleach concentration method
  (e) PCR / Nucleic-acid testing
2. What is your test turn-around time? …………… days/hrs (please circle the appropriate unit)
3. What is the average number of tests you perform monthly?
  (a) Less than 50
  (b) 55–99
  (c) 100–199
  (d) 200–499
  (e) 500 and more
4. (4.1) How many laboratory personnel have been retrained in TB diagnosis between January – December 2008? …………………………….
  (4.2) What kind of training was done?
  (a) In-house
  (b) Local
  (c) International
5. How are samples stored?
  (a) Room temp
  (b) 2–8 °C
  (c) -20 °C
  (d) -70 °C
  (e) Don’t store samples
6. For how long are they stored before analysis? …………………… days
7. (7.1) Have you registered for any external quality assurance programme for TB diagnosis?
  (a) Yes
  (b) No
  (7.2) If yes, which is it? ………………………………………………………… ?
  And what assay? …………………………………………………………………. ?
  (7.3) If no, would you want to register for a national external quality assurance programme?
  (a) Yes
  (b) No
8. What internal quality control measures do you use for your assay(s)?
  (a) Use of both (-) and (+) control smears
  (b) Reagents check (batch & expiry dates)
  (c) Smear size
  (d) Two microscopists concurring
9. (9.1) Do you have OTHER in-house quality control and assurance measures?
  (a) Yes
  (b) No
  (9.1) If yes, list all?
  (a) …………………………………………………………………………………
  (b) …………………………………………………………………………………
  (c) ………………………………………………………………………………….

**Section D – to be completed by head of the malaria unit Malaria diagnosis (Tick all that apply)**

1. Which of these tests do you perform?
  (a) Thin film
  (b) Thick film
  (c) QBC
  (d) Rapid test
  (e) Cyscope
2. What is your test turn-around time? …………… days/hrs (please circle the appropriate unit).
3. What is the average number of tests you perform monthly?
  (a) Less than 50
  (b) 55–99
  (c) 100–199
  (d) 200–499
  (e) 500 and more
4. (4.1) How many laboratory personnel have been retrained in Malaria diagnosis between January - December2008? …………………………….
  (1.2) What kind of training was done?
  (a) In-house
  (b) Local
  (c) International.
5. How are stained films stored?
  (a) On the bench
  (b) Slide box.
6. For how long are they stored before analysis? …………………… days
7. (7.1) Have you registered for any external quality assurance programme for malaria diagnosis?
  (a) Yes
  (b) No
  (7.2) If yes, which is it? ………………………………………………………….
  And what assay? ……………………………………………………………………
8. (7.3) If no, would you want to register for a national external quality assurance programme?
  (a) Yes
  (b) No
9. (8.1) Do you have any in-house quality control and assurance measures?
  (a) Yes
  (b) No
  (8.2) If yes, list all?
  (a) …………………………………………………………………………………
  (b) …………………………………………………………………………………
  (c) ………………………………………………………………………………….
10. Do you find out if malaria drug(s) has (have) been administered before the test?
  (a) Yes
  (b) No

### Phase 2: Provision of proficiency testing service

Of the 106 laboratories that completed the questionnaires, 44 indicated their interest regarding participation in the joint PT programme for the three major diseases of public health importance, namely, HIV, TB and malaria. These laboratories were enrolled in the second phase of the study. By September 2010, pre-characterised panels were prepared for HIV, TB and malaria in the Nigerian Institute of Medical Research (NIMR)’s reference laboratories and were distributed to the participating laboratories by courier. Four rounds of panels each of HIV, TB and malaria were sent to each laboratory on a quarterly basis for a year.

### Characterisation of panels

#### HIV

Blood samples obtained from blood banks were characterised at the national reference laboratory by testing on the Determine™ HIV-1/2 rapid test (Abbott, USA), Genscreen™ Ultra HIV Ag-Ab enzyme immunoassay (Bio-Rad, France) and NEW LAV-BLOT I and NEW LAV-BLOT II western blotting (Bio-Rad, France). The tests were all carried out according to their manufacturers’ instructions.^11,12,13^ Five panels, each comprising three positive and two negative samples, were sent to the participating laboratories for each round. The PT panels were scored based on the HIV-positive or -negative status assigned by the reference laboratory. The correct use of the national testing algorithm was also assessed.

#### Tuberculosis

Following informed consent, fresh sputum specimens were collected from patients who attended the Directly Observed Treatment Short-course (DOTS) clinic at NIMR. Panel slides were prepared as described by Martinez-Guarneros et al.^14^ and each slide reading was carried out by two independent microscopists who arrived at a consensus. A total of five unstained slides per panel was sent to each laboratory (20 slides in total), with instructions to stain panels using the laboratory’s routine procedure to identify and quantify the acid-fast bacilli (AFB) using the WHO or International Union Against Tuberculosis and Lung Disease (IUATLD) grading system. The ratio of positive to negative was varied randomly in each round of panel distribution. The participating laboratories were assessed on correct identification of the slide status and quantification of the AFB, as compared with the assigned characteristics from the reference laboratory.

#### Malaria

Following informed consent, blood was collected in EDTA tubes from malaria parasite-positive patients with varying degrees of parasitaemia, as well as from malaria parasite-negative subjects. Before samples were sent out, thick and thin films were made on the same slide and stained with Giemsa stain at the reference laboratory, according to the standard method.^15^ The films were screened to ensure a good staining reaction. The slides were examined by two independent microscopists for cell distribution, parasite density count, species and stage identification. A limit of 30% was set to reach a consensus; however, where consensus was not reached, a third microscopist read the slide as a tiebreaker. The consensus information was recorded for each slide. For each round, the slides were packaged in the slide boxes in sets of three negatives and two positives. Laboratories were expected to determine the parasite status and density as well as analyse each slide for species identification. Results were assessed based on these parameters and the errors identified. Reports were returned showing error types and suggestions for improvement.

### Panel distribution

For each shipment, the panels were parcelled in a triple packaging format, including instructions and reporting forms. The panels were sent in cold boxes through a courier agent to each zonal coordinator in order to save cost. Each report form contained sections for results and comments, enabling participants to provide feedback. The zonal coordinators were responsible for distributing the panels to the participating laboratories within their zone. Participants were instructed to return results within two weeks of receipt of the panels and the same channel was then used for returning the results and feedback forms to NIMR. Returned results were assessed and scored based on performance as compared with the expected results and individual performance scores were then returned to the participants through the same zonal coordinators.

### Mentoring component

After the first round of panel distribution, job aids (i.e., brief procedural instructions) for diagnosis of each disease type were prepared and sent to all the laboratories to help improve performance. At the end of the second round of panel distribution, because of cost constraints, 13 laboratories were recommended for personnel retraining as a result of poor performance. These laboratories were invited for a fully-sponsored training course at NIMR. Personnel from 11 of the 13 laboratories attended the week-long training, during which participants spent two days each on practical sessions on the laboratory diagnosis of TB and malaria and one day on HIV diagnosis. The training also consisted of didactic sessions on QMS. Panels were sent out to the laboratories for the third round immediately after the training. There was no mentoring session before the fourth round.

### Data analysis

Results were scored based on the assigned PT provider ratings and characteristics. Discordant results were assigned zero points, whilst concordant results were assigned 20 points per sample, for a total of 100 points per panel. Scores of 80% and above were reported as satisfactory, which is the generally-accepted standard.^16^ An unassigned score for a particular distribution indicated that a laboratory did not return the result. All scored results were entered into a FileMaker Pro v10 database where individual reports were generated for each laboratory. The data were then exported and analysed in a Microsoft® Excel spreadsheet. The feedback from the laboratories was analysed and suggested improvements for the PT services were implemented where possible.

### Ethical considerations

Ethical approval was obtained from the Institutional Review Board of the NIMR (11 May 2009). Only those sites that indicated an interest in participating were enrolled in the study, at no cost. Laboratories were free to decline participation in the study at any point in time.

## Results

### Questionnaire survey

Table 1 shows the results of the surveyed laboratories from the first phase of the study. This study used an abridged grading system of the WHO Stepwise Laboratory Quality Improvement Process Towards Accreditation (SLIPTA) to assess laboratories' adherence to the International Organization for Standardization (ISO) 15189 standard, measuring laboratory quality on a scale of zero to five stars.^17^ The laboratories attained, based on self-reporting, an average score of 65%, which is equivalent to two stars out of five. It was also found that of the 106 laboratories that completed the questionnaire, 68 (64%) reported having a system of result validation and only 34% (*n* = 36/106) provided scheduled maintenance of equipment. Most of the laboratories did not have preventive maintenance policies, thereby possibly undermining the quality of results generated by the equipment. Internet access was found to be uncommon (*n* = 36/106; 34%) amongst the respondents. The survey also showed that very few laboratories were registered for PT for HIV (*n* = 35/106; 33%), TB (*n* = 33/106; 31%) and malaria (*n* = 19/106; 18%). Forty-four (42%) of the 106 laboratories indicated interest in participating in the PT for the three diseases offered in this study as they had not been registered for PT previously.

**TABLE 1:** Survey results of the 106 surveyed laboratories.

Quality characteristic Number of	laboratories that responded positively	Percent of laboratories that responded positively (%)
**Documents**		
Standard Operating Procedures (SOPs)	99	93
Infectious waste disposal guidelines	95	90
**Purchasing and inventory**		
System for evaluation and selection of suppliers of kits	88	83
Supply forecast and inventory management	79	75
**Process control**		
Sample referral	75	71
Registration in any proficiency testing for:	35	33
- HIV	33	31
- TB	19	18
- Malaria		
**Information management**		
System for validating results	68	64
Storage of results electronically / in registers	101	95
Duration of storage of results:		
- Years	84	83
- Months	14	14
- Days	3	3
**Equipment**		
Back-up generator	100	94
Preventive maintenance agreement	36	34
Reliable internet service	36	34

Overall average of 65% (2 stars) based on the World Health Organization Stepwise Laboratory Quality Improvement Process Towards Accreditation Checklist.

### Participation and response rate of laboratories

Of the 44 laboratories that indicated interest in participating in the joint PT programme, 10 (23%) were publicly-owned laboratories within hospital settings whilst 34 (77%) were private, stand-alone laboratories. In the second phase of the study, two laboratories opted out, one at the first round of panel distribution and the other after the second round of distribution. A few laboratories did not return results and were thus not assigned scores. Laboratories that did not receive scores included 3/44 (7%) in the first round, 5/43 (12%) in the second, 2/42 (5%) in the third and 7/42 (17%) in the fourth round. On average, 10% failed to return results on one or more PT samples.

### Proficiency testing panel testing results

**FIGURE 1:**
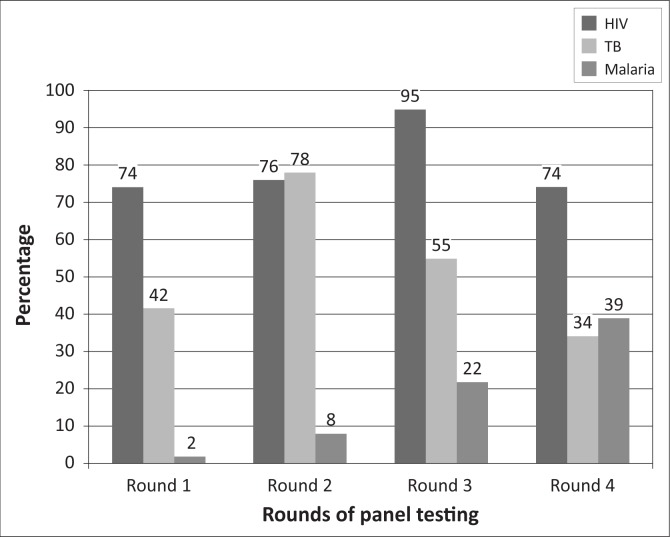
Average performance of laboratories at different rounds of panel testing.

**FIGURE 2:**
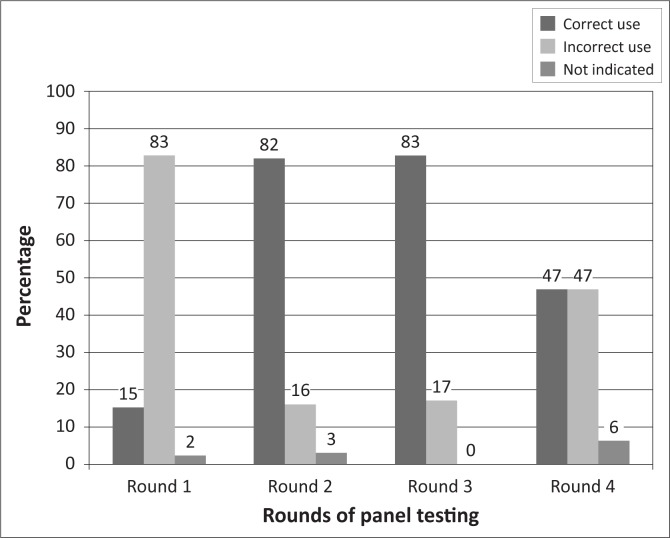
Use of national testing algorithm in HIV diagnosis.

Most laboratories returned the PT panel results within the two weeks stipulated, but the time had to be extended for some laboratories because of political insecurity. All the laboratories tested the HIV panel using rapid test kits, either serially or in parallel algorithms. Some did not confirm a positive result with a second test kit. The PT results showed improvement in HIV PT scores from an average of 74% in the first round to 95% in the third round, but this was not sustained in the fourth round (Figure 1). The major issue observed with HIV PT was the incorrect use of the national testing algorithm, which requires consistent results from two rapid test kits before confirming a positive HIV status; some laboratories used only a single reactive result (Figure 2).

**FIGURE 3:**
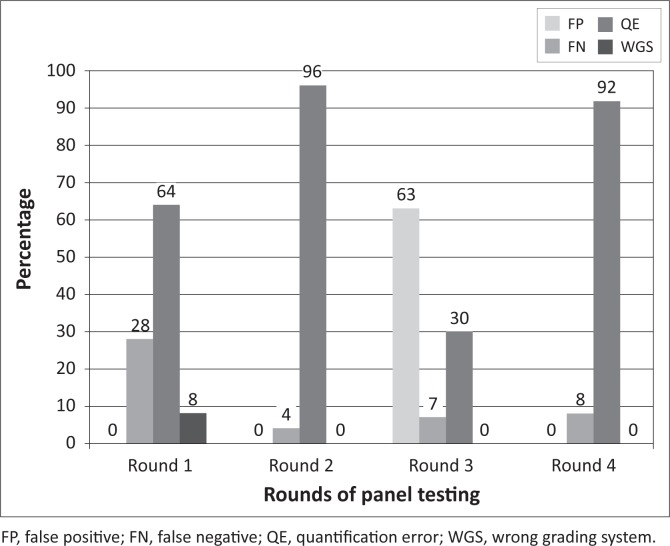
Comparison of errors in tuberculosis diagnosis.

For TB PT, all laboratories stained the slides using the Ziehl-Neelson staining technique. There was an improvement after the first round from an average score of 42% to 78% in the second round; however, the average dropped consistently from that point to 34% in the fourth round (Figure 1). The issues observed with TB PT included quantification errors and a high level of false negative results (Figure 3). An unusually high level of false positives was observed in the third round of the PT.

**FIGURE 4:**
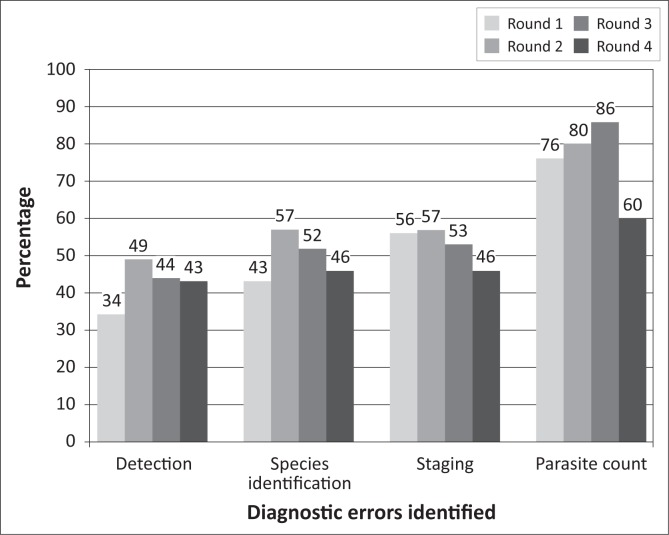
Frequency and types of errors identified in malaria diagnosis.

Although performance in malaria PT appeared poor, there was a steady increase in average scores from 2% in the first round to 39% in the fourth round (Figure 1). Participants appeared to continue to have difficulties with parasite detection and count throughout the testing period (Figure 4). One laboratory confirmed the use of rapid test kits for malaria; its results were not included in the analysis.

### Feedback from participating laboratories

**FIGURE 5:**
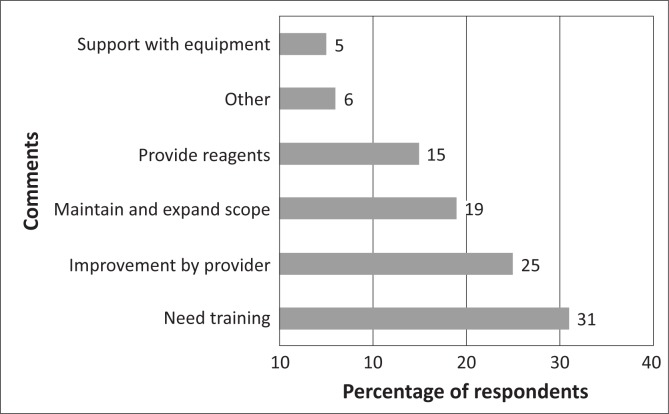
Categorised comments by participating laboratories.

A total of 81 persons provided feedback throughout the duration of the PT. The respondents were at liberty to express their concerns to NIMR on any issues whatsoever. Figure 5 shows the categorised comments from the participating laboratories.

There was a great demand for training expressed by 25 (31%) of those who gave feedback. Some requested on-site mentoring visits, whilst others wanted practical training sessions organised by the PT provider. Some stated that training would enhance their competence and serve as a motivating factor for participation. Twenty (25%) of the feedback responses provided useful suggestions to the provider for improvement. Some requested that the time to return results be extended, whilst others requested that the quarterly exercise be replaced by samples being distributed every 2 months. Fifteen (19%) respondents also requested the continuation of the programme and expansion of the scope to include more analytes for laboratory investigations of other diseases, aside from the HIV, TB and malaria, as well as inclusion of more laboratories. Whilst 12 (15%) of the respondents requested the provision of reagents for PT, four (5%) complained about bad microscopes and needed some assistance, either financially or through direct provision of better microscopes and supervisory visits.

A number of comments were actionable immediately and helped the provider to improve the quality of PT samples. The most prominent example was a series of complaints of leakage of plasma samples, which the provider responded to by changing the sample tubes. Equally important complaints after the first round were broken slides and the quality of some of the malaria parasite slides. The provider improved on the packaging by ensuring proper sealing and positioning of the slide boxes so as to prevent damage during transportation. To address the slide quality, three or four readers reviewed the stained slides for subsequent panels for the second, third and fourth rounds and the best slides with the lowest inter-reader variability were selected and sent. An expert on malaria panel preparation from the National Research Centre, Burkina Faso, where the staff had previously acquired malaria panel preparation skills, visited the provider to ensure quality practices.

## Discussion

The initial phase of the study indicates the prevalence of a poor culture of QMS in Nigerian laboratories. From the questionnaire survey, the laboratories earned an average of two stars, despite the fact that the grade was attained by self-reporting and not by an external audit. The lack of QMS culture creates concern regarding the accuracy, reliability and timeliness of clinical results generated in laboratories. This finding supports previous observations that, in sub-Saharan Africa, laboratory infrastructure and personnel are affected adversely by the lack of resources and prioritisation, hampering laboratory system efforts in the fight against infectious and chronic diseases.^4,5^ As a result, the accessibility of laboratory testing and the quality of available services remain a serious challenge.^7^ There is a dire need to create a culture of quality management in Nigerian laboratories, which will help practitioners appreciate the necessity of results validation and participation in PT and other programmes. NIMR plans to address this need with its newly-awarded training grant to build the culture of QMS in both private and government-owned laboratories. This grant utilises the Strengthening Laboratory Management Toward Accreditation (SLMTA) programme to develop capacity for laboratories not supported by the US President’s Emergency Plan for AIDS Relief (PEPFAR) funds

A lack of resources as well as a poor understanding of the benefits of participation in an external PT programme may have been responsible for the low enrolment in this study. Of the laboratories that did enrol, two private laboratories opted out of the PT programme after commencement. Without any formal communication, the first laboratory was closed down at the point of delivery of a set of panels. The closure may have been related to the political crises in that region. The second laboratory communicated to the provider that the staff would no longer be able to participate in the study because the laboratory owner had gone back to school for full-time study.

Investing in QMS is expensive and time consuming. As such, care should be taken in the selection of the laboratories enrolled in such PT projects in order to ensure their ability and willingness to provide the needed services. The rate of failure to return results varied despite the fact that the due dates for the results were extended at the request of some of the participating laboratories. One reason for this variation could possibly be a poor understanding of the importance of PT programmes. Some of the laboratories’ staff members reported that they had to wait for the most senior laboratory scientist, often the owner of the laboratory, to be present during sample analysis. Training will help improve understanding and increase participation.

The incorrect use of the national HIV testing algorithm was common at the outset of this study. The nationally recommended algorithm includes serial testing, which requires a second test for an initial reactive sample. Some laboratories used test kits outside the nationally-approved kits; some used a single test to confirm HIV infection; and others used parallel testing algorithms. Correct use of the standardised HIV testing algorithm would reduce the risk of issuing false reactive results. When so much time and so many resources are spent on developing HIV testing algorithms, it is essential that countries ensure their proper dissemination to all levels where HIV testing is carried out. In this study, provision of a job aid with step-by-step instructions for HIV testing after the first round of panel distribution resulted in marked improvements. However, some laboratory scientists complained that their job aids were kept in the office of the head of the laboratory and not at the point of use in the laboratory. Lack of available job aids in the laboratory may have affected the laboratories' performance in this study.

Provision of job aids may also have contributed to the improvements observed in the diagnosis of TB in the second round of panel distributions; however, this improvement was not sustained and quantification errors were common. This finding underscores the challenges of managing TB patients on therapy, as the efficacy of therapy would be difficult to monitor. Other error types that could have a serious impact on the community were the high rate of false positive results observed in the third round and the persistent false-negative results observed throughout the study. These errors have serious implications: individuals diagnosed incorrectly as positive will most likely be placed on unnecessary therapy, whilst individuals who are diagnosed incorrectly as negative will be released into the community and will spread the infection. All measures must be put in place to halt this trend, especially as Nigeria has been ranked 13th on a list of 22 countries with the highest burden of TB.^16^

Unlike the positive performances reported in an eight-year EQA of public reference laboratories, which recorded 82% acceptable results for malaria species identification,^18^ this study observed a comparatively low rate of acceptable malaria results. The poor performance observed for both TB and malaria diagnoses may be connected to the report in Phase 1 of the study, in which only 34% of the laboratories reported having preventive maintenance for their equipment. It is important that preventive maintenance programmes be established in laboratories in order to ensure proper functioning of equipment so as to guarantee the accuracy and reliability of test results. Confirming the need for better equipment maintenance, 5% of respondents complained about the quality of their microscopes. Inadequate equipment maintenance, as observed in this study, is not limited to Nigeria; poor maintenance culture is one of the major challenges in strengthening health systems in sub-Saharan Africa.^19,20^ In spite of equipment limitations, there was continual improvement in performance on malaria panels, particularly with regard to parasite count and staging. This progression gives hope for improvement in the diagnostic proficiency of malaria microscopists if more efforts can be devoted to their training. To bridge this gap, NIMR now provides annual training for malaria microscopists from all over the country.

Study participants demonstrated that they recognise that there are gaps in their knowledge and are willing to be trained for improvement. Thirty-one per cent of the participants who gave feedback from Phase 2 of the study requested further professional training. Moreover, some of the participants who attended the resulting training commended the organisers, as they had not undertaken any prior in-service training. Efforts directed at in-service training should be increased and extended to private laboratories that contribute a great deal to the health system, particularly in Nigeria.

Those who had not participated in a PT programme previously also requested that the programme be sustained and expanded with regard to the scope of analytes and the number of participating laboratories. This request came despite the varying acceptable result rates obtained from the laboratories. Such feedback is indeed a clarion call for more donor investment in EQA.

It is essential that individual African countries be strengthened in order to take up the challenge of providing EQA in their respective countries, in order to extend PT programmes to these laboratories. Currently available programmes are accessible to national public health or reference laboratories in Africa, but do not benefit peripheral or private laboratories. Access to PT programmes will also motivate the drive toward accreditation, as observed in South Africa.^7^ The quest for accreditation can help laboratories address most other concerns. Preparing for accreditation helps to strengthen laboratory management in and application of best practices throughout the laboratory system. Awareness of laboratory accreditation is gathering momentum at present in Nigeria, as is evidenced by the enrolment of 30 laboratories for accreditation preparedness training, the preparation for enrolment by others and the high demand for training by still more laboratories. There are currently 30 personnel who have been trained to roll-out the SLMTA programme in Nigeria, three of whom qualified as master trainers;^21^ several other in-country training courses to prepare laboratories for accreditation are on-going.

In summary, this study identified gross deficiency in the quality of laboratory services rendered across the country, indicating a poor state of QMS. The PT study was well received, with demands to extend its scope. Although most participants requested training and on-site mentoring, the training provided by this exercise was too short and did not have the desired impact for HIV and TB diagnoses. Nevertheless, just as it has been reported from regional EQAs that public health and reference laboratories in the African region are capable of the accurate determination of disease status,^18,22^ so it is believed that the laboratories that participated in this study also can perform satisfactorily if given the necessary support. For example, similar laboratories in Uganda^23^ and other resource-constrained countries^24^ have been supported in their endeavours to improve the quality of their services and have yielded remarkable improvements. There is, therefore, a need to strengthen laboratory systems in individual countries by providing PT programmes to clinical laboratories, including those that are privately owned with high volumes of work. The implementation of PT programmes will enhance the drive toward laboratory accreditation in the region.

### Limitations of the study

Differences may have arisen from the self-administered questionnaires that could have influenced the findings reported in this study. Also, there may have been varied readings by microscopists for the blood and sputum films, affecting study findings.

### Conclusion

There was gross deficiency in the implementation of QMS which inadvertently affected the proficiency of the laboratories in the diagnosis of HIV, TB and malaria. Concerted efforts are therefore required to train Nigerian laboratories on QMS, which would yield the desired outcome as observed from this study.
